# High-performance vector bending and orientation distinguishing curvature sensor based on asymmetric coupled multi-core fibre

**DOI:** 10.1038/s41598-020-70999-8

**Published:** 2020-08-20

**Authors:** Oskar Arrizabalaga, Qi Sun, Martynas Beresna, Timothy Lee, Joseba Zubia, Javier Velasco Pascual, Idurre Sáez de Ocáriz, Axel Schülzgen, Jose Enrique Antonio-Lopez, Rodrigo Amezcua-Correa, Joel Villatoro, Gilberto Brambilla

**Affiliations:** 1 Department of Communications Engineering, University of the Basque Country (UPV/EHU), Ingeniero Torres Quevedo s/n, 48013 Bilbao, Spain; 2 Optoelectronics Research Centre, University of Southampton, Southampton, SO17 1BJ UK; 3 Fundación Centro de Tecnologías Aeronáuticas (CTA), Miñano, Spain; 4 CREOL, The College of Optics and Photonics, University of Central Florida, Orlando, USA; 5 IKERBASQUE-Basque Foundation for Science, 48011 Bilbao, Spain

**Keywords:** Engineering, Optics and photonics, Physics

## Abstract

Fibre optic technology is rapidly evolving, driven mainly by telecommunication and sensing applications. Excellent reliability of the manufacturing processes and low cost have drawn ever increasing attention to fibre-based sensors, e.g. for studying mechanical response/limitations of aerospace composite structures. Here, a vector bending and orientation distinguishing curvature sensor, based on asymmetric coupled multi-core fibre, is proposed and experimentally demonstrated. By optimising the mode coupling effect of a seven core multi-core fibre, we have achieved a sensitivity of − 1.4 nm/° as a vector bending sensor and − 17.5 nm/m^−1^ as a curvature sensor. These are the highest sensitivities reported so far, to the best of our knowledge. In addition, our sensor offers several advantages such as repeatability of fabrication, wide operating range and small size and weight which benefit its sensing applications.

## Introduction

Mechanical structures are becoming more and more complex with the introduction of intricate geometries and composite materials^1^. Fibre reinforced polymers are composites used in almost every type of advanced engineering structures, with their usage ranging from aircraft, helicopters and spacecraft through to boats, ships and civil infrastructure such as bridges and buildings. They are made with reinforcement fibres among the several types of composites^2,3^ that are embedded in a polymer resin (mostly epoxy).

Composite materials represent a growing piece of the aerospace material pie. They reduce weight and increase fuel efficiency while being easy to operate, design, shape, and repair. Thus, the study of the mechanical behaviour of these structures has enormous significance. In order to obtain the required level of performance for flight structures, detailed knowledge of material limitations, structural stability and strength aspects is required. Key sources of information on the mechanical performance are sensors based on electronic technology^4–6^. Some of the benefits of this type of sensor include accuracy, a wide variety of sizes and shapes, and a simple operating principle. However, they also have several critical shortages. Their performance is affected by humidity, temperature and hysteresis, repeatability and accuracy fall with prolonged use, and also they can be damaged by statics or current overloads. Another constraint is that they cannot work in the presence of electromagnetic fields.

Over the last few years, optical fibre sensors (OFSs) have emerged as an alternative to their electronic equivalents in the process of testing material limitations for engineering structures^7,8^. Fibre-based sensors can be used to measure strain, temperature, pressure, bending and other quantities^9,10^. Such sensors are normally interrogated by coupling the light from a laser (often a single-frequency fibre laser) or from a superluminescent source. The light propagating in the fibre changes its parameters such as spectral composition, intensity, polarization, which can be easily analysed and quantified by means of an optical spectrum analyser (OSA) or power meter.

OFSs in general offer some big advantages over their electrical equivalents such as their immunity to electromagnetic interference and high voltage, low hazard potential, immunity to lightning strikes, capability of remote sensing and small size, just to mention a few. All described features make them very attractive, e.g. in the study of material limits in aeronautical structures^11^.

Many fibre-based technologies are being developed but commercial solutions mainly boil down into two categories: point sensing where the active part is a short segment of fibre^12^ and distributed sensing where the entire fibre operates as the sensor^13^.

Fibre Bragg grating^14^ (FBG) sensors are by the far the most widely fibre-based technology used as a point sensor. They are settling heavily in the industrial market and their range of applications continues to grow. However, thermal and transversal strain sensitivities, high-cost for building and maintaining, limited supplies and the difficulty in demodulating wavelength shift are their main drawbacks of this approach^15,16^.

In order to enhance the sensitivity in the last few years, different fibre-based sensors schemes have been proposed. An FBG in a waveguide-array microstructured optical fibre^17^, a long-period fibre grating in an index-guiding photonic crystal fibre^18^ or Fabry–Perot interferometers^19,20^ (FPIs) are some examples. Of these, the latter offer the higher sensitivity^21^. Nevertheless, due to the air-cavities of FPI structures, the devices have a low mechanical strength. Therefore, the previously reported techniques have the inconvenience of a low sensitivity or weak mechanical strength.

An alternative approach is to use special optical fibres that contain multiple cores, which may e.g. be arranged on a ring around the fibre axis or on six cores on the edges of a hexagon and a central core in addition. Such fibres are called multi-core fibres (MCF)^22^. In principle, each of the fibre cores in such a fibre can act as a separate waveguide, so light can independently propagate through those cores. However, if the distance between the cores is small, mode coupling between the cores takes place because the corresponding mode fields have a significant spatial overlap^23^. That is to say, the light which is initially coupled into one core can eventually be coupled over to other cores. The exchange of light between the cores is extremely sensitive to external influences such as strain or temperature changes. In fact, several authors have already proposed various types of schemes to develop sensors with MCF in order to achieve sensors with functionalities superior to those based on conventional fibres^24,25^. Nevertheless, although MCF-based sensors have been already demonstrated^26–29^, we believe that the coupling mode effect in strongly coupled MCFs has not yet been fully exploited. For instance, the MCF used in the previously reported works was fabricated with an identical index for all cores and with symmetric geometry. This fact restricts efficient exploitation of the mode coupling between cores because the effect is not sensitive to complementary actions e.g. distinguishing curvature orientation.

In this work, we report on the MCF-based sensor capable of distinguishing curvature orientation. We break the symmetry of a MCF by using femtosecond laser writing to modify the index of one of the six surrounding cores of a seven core fibre, which are symmetrically arranged around a central core. The absence of the symmetry enabled us to implement a sensing device capable of detecting the orientation as well as magnitude of the curvature with high sensitivity. Taken into account the fibre optics advantages mentioned above, we believe that our proposed device is suitable to be used in the study of mechanical behaviour of e.g. aeronautical structures. In fact, our sensor was evaluated in a static test of an aeronautical component, made of carbon fibre. In such test, it behaved in the same way that a strain sensor with which it was compared.

## Results

**Figure 1:**
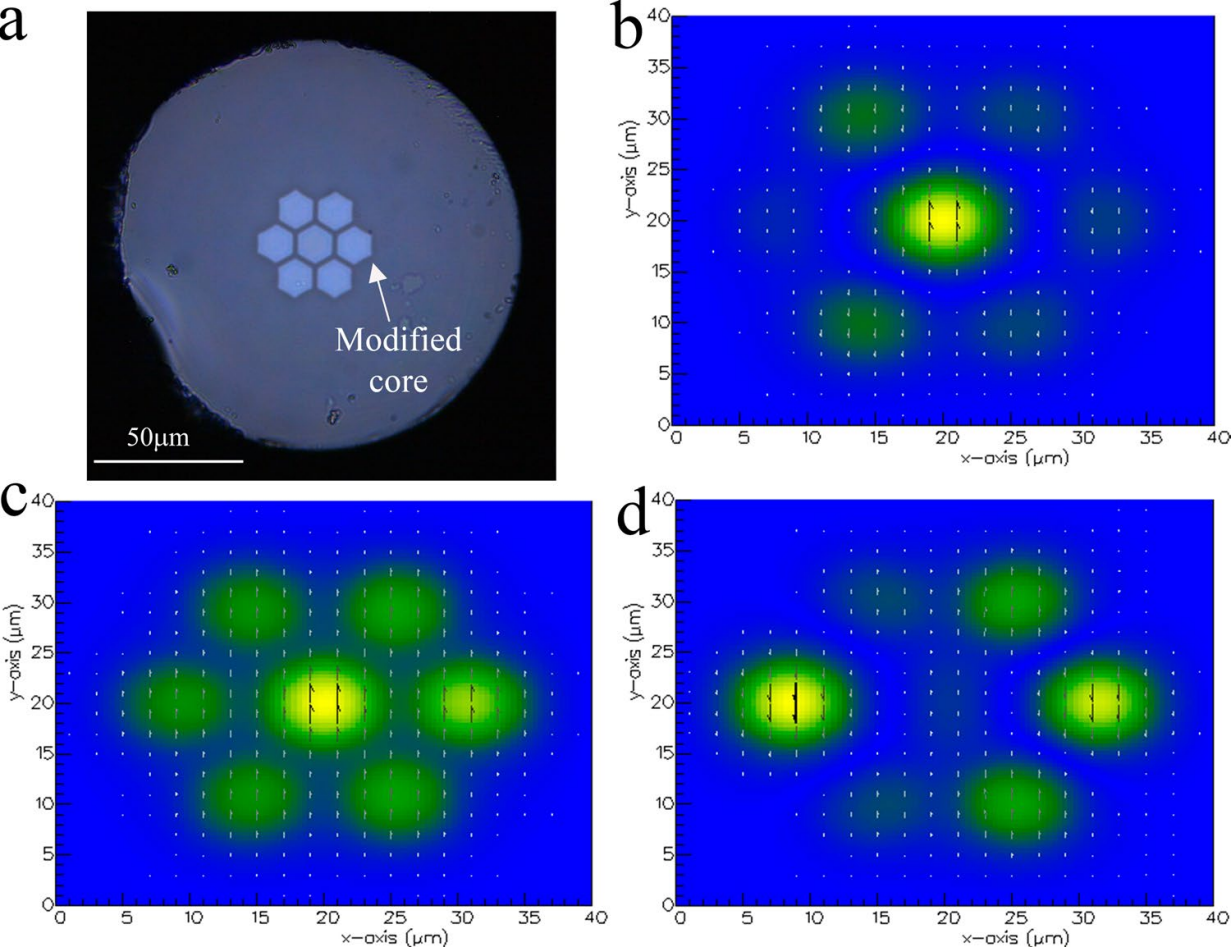
(**a**) Micrograph of the cross section of the MMCF. (**b**,**c**) Simulated fundamental mode field profile, and higher order mode with a central peak, respectively. (**d**) One of the five *y*-polarised higher order modes without central peak. In the simulations, the MMCF was excited at 1,550 nm and the colours represent the amplitude of the field.

Our proposed sensor is based on a strongly-coupled multi core fibre (MCF) fabricated at the University of Central Florida (Orlando, USA) by the well-established stack and draw method. The MCF consists of a symmetrical structure formed with a central core surrounded by another six cores made of silica doped with germanium and embedded in pure silica cladding (see Fig. 1a). The numerical aperture (NA) of the cores at λ = 1.55 μm is 0.14, matching with a standard SMF. The radius of each core (*r*) is 4.5 μm with a core-to-core pitch (*d*) of 11 μm.

The sensing mechanism is based on breaking the refractive index symmetry around the central core. One of the external cores is modified by means of femtosecond laser direct writing to increase the refractive index of a selected volumetric region within the fibre. The laser pulses permanently change the refractive index of the irradiated zone, thus detuning coupling conditions with the neighbouring cores. The dimensions of the inscribed structure were 10 μm × 10 μm in cross section and 4 cm in length.

To analyse the propagation of light guided in the MMCF, the inter-core coupling behaviour can also be interpreted as the beating of supermodes. From a full vector finite-element mode solver (Photon Design, Oxford, UK) it has been found that our modified multi core fibre (MMCF), in the range from ~ 1,250 to ~ 1,650 nm, supports seven super-modes each of which has two orthogonal linear polarizations (*x* and *y* polarizations). They can be divided into three categories: fundamental modes, higher order modes with a central peak and higher order modes without central peak (see Fig. 1). We have labelled them as SM_1_, SM_2_ and SM_3_, respectively.

**Figure 2:**
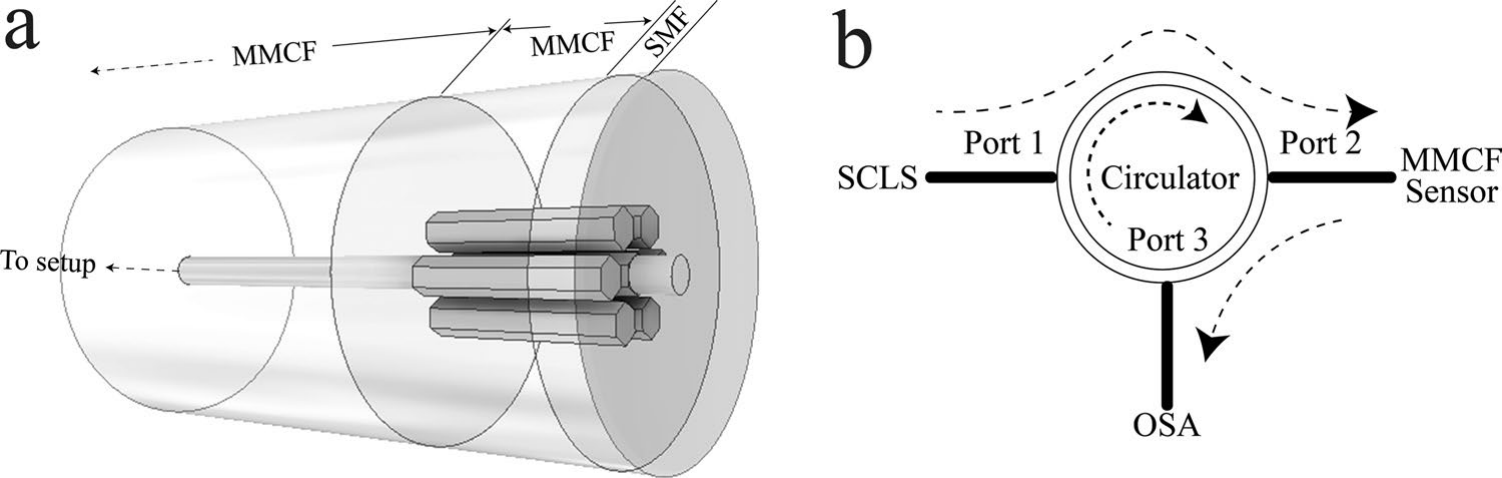
(**a**) Illustration of the structure of the MMCF sensor device. (**b**) Experimental setup. *SCLS* super continium light source.

The structure of our proposed sensing device consists of two single mode fibres (SMF), spliced axially aligned, at both the ends of a short segment of MMCF of length *L* as can be seen in the Fig. 2a. Thereby, when the fundamental mode of the SMF (HE_11_) is injected from the input-SMF to the central core of the MMCF, the SM_1_ and SM_2_ will be excited due to large nonzero modal overlap with the input mode. The SM_3_ modes are not present, or their power is so low that they can be neglected. The SM_1_ and SM_2_ will beat with each other as they travel through MMCF, leading to a periodic coupling spectrum which can be observed via the output SMF.

To study the power in only the central core, note that the mode coupling between all the cores of a MMCF is given by a set of coupled mode equations^30–32^. Then, when the fundamental mode of the SMF is launched into central core of MMCF, the theoretical solution for the field amplitude in the central core at distance *L* is given by^33,34^:

1 $$ A(L) = \left[ {Cos\left( {\sqrt 7 CL} \right) + \frac{j}{\sqrt 7 }Sin\left( {\sqrt 7 CL} \right)} \right]e^{ - jCL} $$

The *C* parameter denotes the coupling coefficient between adjacent cores and it can be approximated from their modal field spatial overlap to give:

2 $$ C = \frac{\lambda }{{2\pi n_{co} }}\frac{{U^{2} }}{{r^{2} V^{2} }}\frac{{K_{0} \left( \frac{Wd}{r} \right)}}{{K_{1}^{2} \left( W \right)}} $$

where $U = r\left( {k_{0} n_{co}^{2} - \beta^{2} } \right)^{1/2} ,\,W = r\left( {\beta^{2} - k_{0} n_{cl}^{2} } \right)^{1/2} {\text{and}}\,\,V = rk_{0} \left( {n_{co}^{2} - n_{cl}^{2} } \right)^{1/2}$; *K*_0_ and *K*_1_ are modified Bessel functions at order m (m = 0, 1); *n*_co_ and *n*_cl_ are the effective refractive indices of the core and the cladding, respectively; k_0_ = 2π/λ represent the wavelength wave number and *β* denotes the propagation constant of each core without mode coupling.

**Figure 3:**
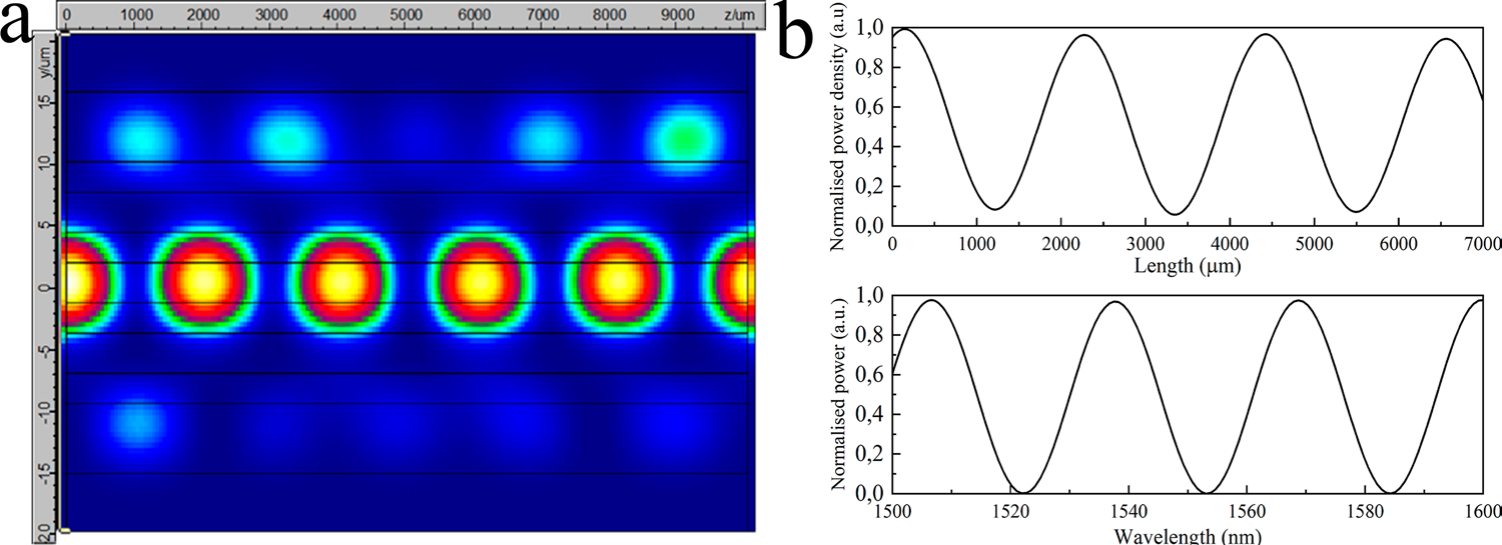
(**a**) 1 cm MMCF field profile simulated by finite-element mode solver software. The centred core was excited at 1550 nm. (**b**) At the top: theoretically obtained normalised power density along 7 mm of the centred core of a MMCF excited at 1,550 nm. At the bottom: spectrum for the centred core at a length *L* = 4 cm.

The normalised power of the mode is defined as *P*_A_ =|A|^2^, which is periodic in *L*. In the Fig. 3a and at the top of the Fig. 3b it can be seen the theoretical simulation (in Photon Design) of the power distribution along a length (*L*) of MMCF when its centered core is excited at 1,550 nm. This power at a length (*L*) can be expressed mathematically as:

3 $$ P_{A} (L) = \frac{1}{7} + \frac{6}{7}Cos^{2} \left( {\sqrt 7 CL} \right) $$

According to Eq. (3), the transmission power of the central core reaches a maximum when the phase condition satisfies (7^1/2^)*CL* = *mπ* (*m* is a positive integer). Therefore, the corresponding wavelength with maximum power (λ_m_) will be located at:

4 $$ \lambda_{m} = \frac{2m\pi }{{\sqrt 7 \frac{{\partial C_{\left( \lambda \right)} }}{\partial \lambda }L}}\quad With\,\,m = \, 1, \, 2, \, 3, \, \ldots $$

At the bottom of the Fig. 3b, we show the theoretical simulation of the power at a length *L* = 4 cm for a wavelength range between 1,500 and 1,600 nm.

In the case of the bent MMCF, the fibre elongates/compresses at the circular bend and the local index changes because of stress-optic effect upon bending. The index transformation is given as follows^22,34^:

5 $$ n^{\prime}(x,y) = n(x,y)\left[ {1 - \frac{{n(x,y)^{2} \,x\,}}{2R}\left( {P_{12} - \nu \left( {P_{11} + P_{12} } \right)} \right)} \right]\,\exp \left( \frac{x}{R} \right) $$

with *n*(x,y) being the refractive index of the undisturbed MCF, *P*_11_ and *P*_12_ are components of the elasto-optical tensor, υ is Poisson’s ratio, *R* is the radius of curvature and *x* is distance from centre of fibre. This transformation allows a circularly curved MCF segment to be modelled as an equivalent straight one.

Bending causes a different percentage of each supermode to be excited and some of the SM_3_ may also become present in the central core. Another consequence induced by the bending is that some higher order supermodes won’t be supported or will have high bending losses. As the change in mode profile also alters their effective index, the power distribution along of the central core of the MMCF will be also affected. Consequently, the spectral peak wavelengths described by Eq. (4) will shift (as well as change in power), providing a measurable quantity for evaluating the bend.

**Figure 4:**
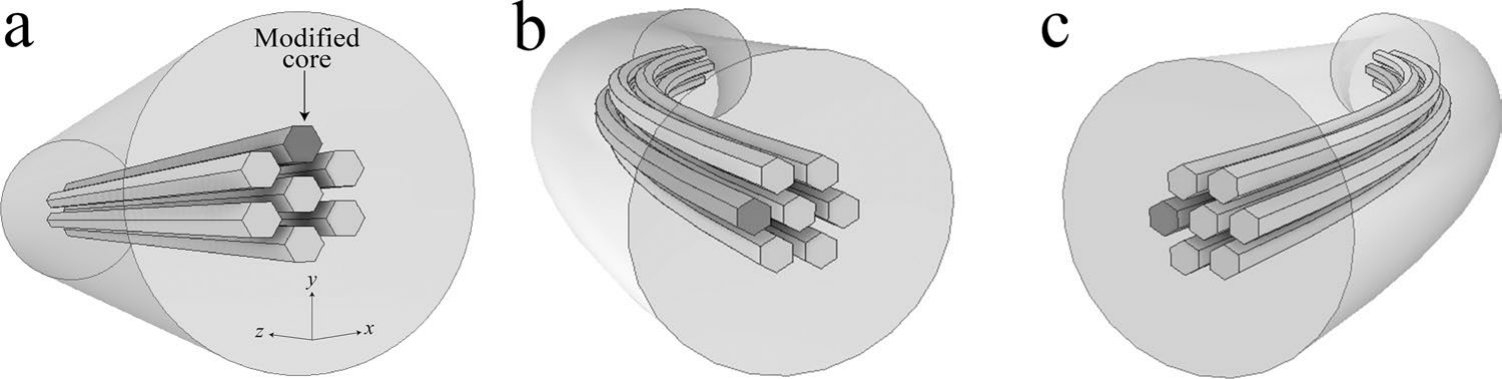
(**a**) Illustration of the distribution of the cores of the MMCF when the index profile is simmetrical respect to the bending plane. (**b**,**c**) Illustrations when the MMCF is rotated 90° with respect to the reference. The index profile is asymmetrical with respect to the bending plane and it varies depending if the written core experiences tension or compression.

Our device was tested in two different configurations; as a vector bending or a curvature direction sensor. First, it was evaluated as a vector bending sensor. The sensor was prepared using a 4 cm segment of MMCF. Then, the written core was placed in such a way that the index of the surrounding cores was asymmetrical with respect to the bending plane (*y,z*) (Fig. 4a). This configuration of the fibre leads to the different excitation and beating of supermodes for bending in the + *y*-direction (see Fig. 4b) compared to the –*y*-direction (see Fig. 4c). Therefore, according to the Eq. (4), λ_m_ will shift to shorter or larger wavelength depending on the bending direction.

**Figure 5:**
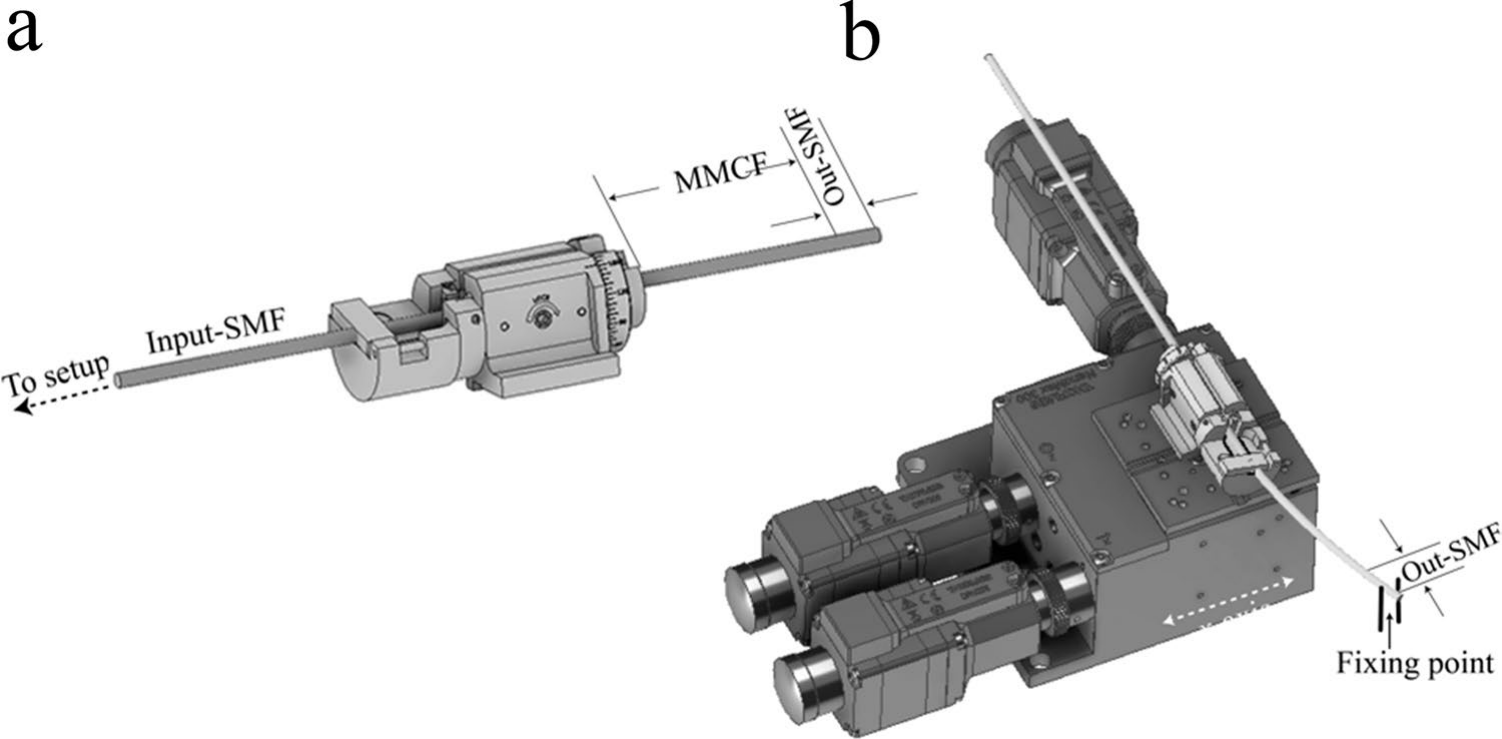
(**a**) Illustration of the precision fibre rotator used to rotate the MMCF. (**b**) Setup used to find the correct position of the cores.

**Figure 6:**
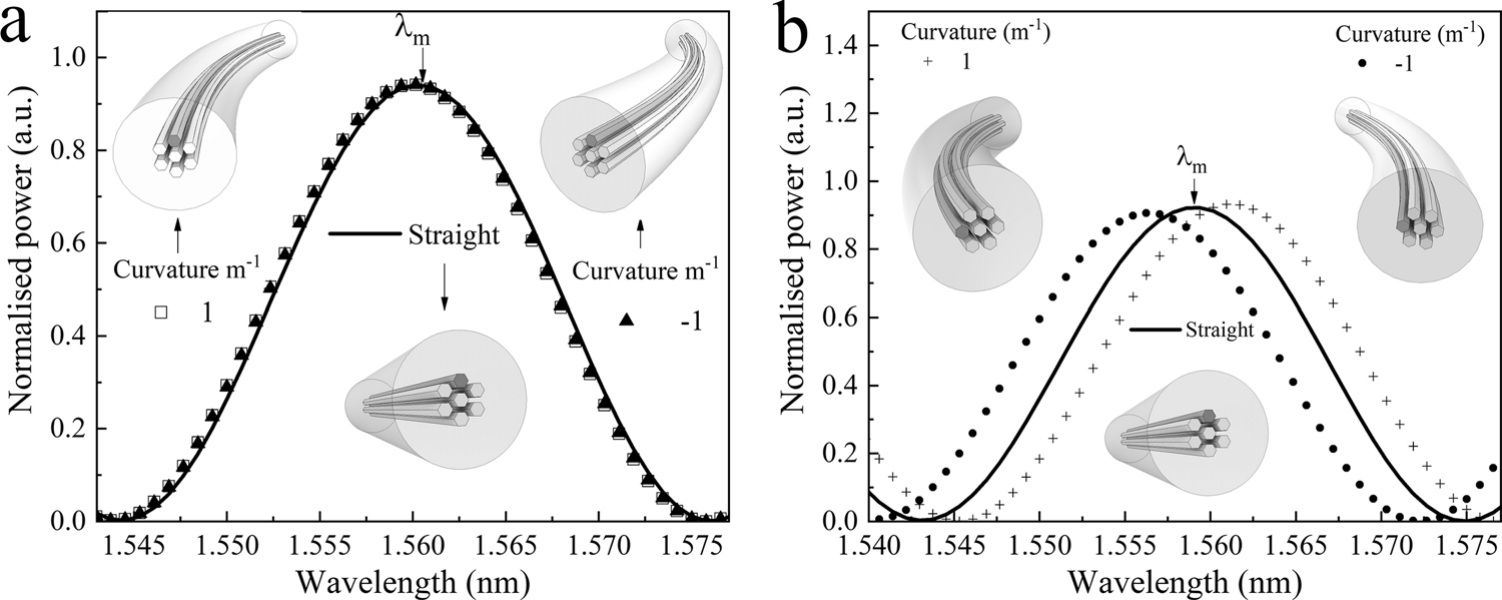
(**a**) Theoretical spectrum of the MMCF device when its index profile is symmetrical with respect to the bending plane. (**b**) The same but with the index profile asymmetrical with respect to the bending plane.

**Figure 7:**
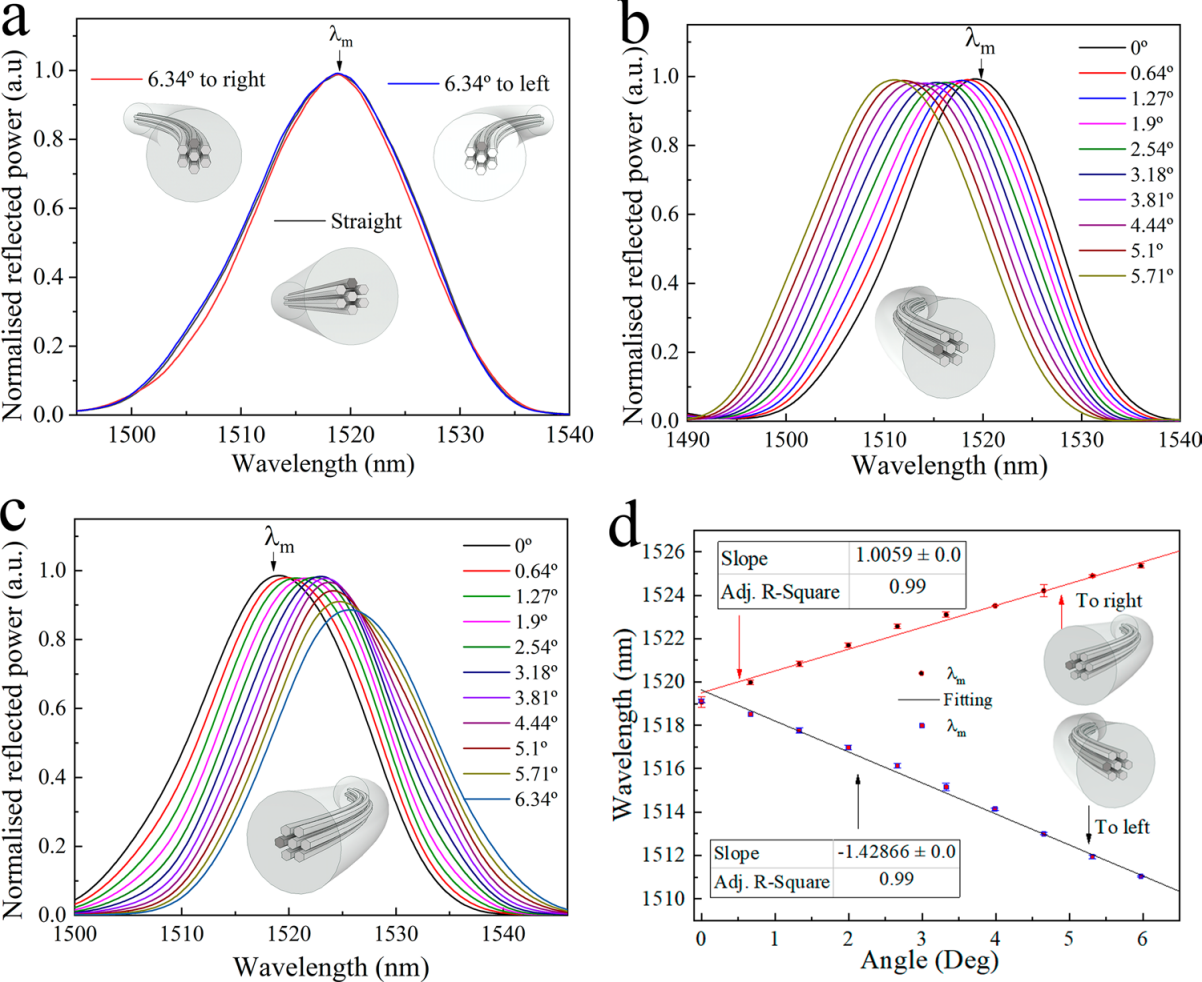
(**a**) Experimental vector bending spectrum of the MMCF device with symmetric index profile with respect to the bending plane. (**b**,**c**) The same but with asymmetric index profile. (**d**) Peak wavelength versus angle for both directions of vector bending.

To place the written core in such position, first, the input SMF was connected to the experimental setup (see Fig. 2b) and the collected spectrum was analysed with an OSA (Yokogawa AQ6370D). Afterwards, the input-SMF was secured to a precision fibre rotator leaving the segment composed of MMCF-out SMF in free space (Fig. 5a). The whole assembly was fixed to a flexure precision stage with a stepper motor actuator (Fig. 5b). The next step was to place the fibre with the written core in a symmetrical position with respect to the bending plane as it is shown in the Fig. 4a. To do this, the output SMF was fixed and translated in the ± x-axis with the flexure precision stage. Simultaneously, the fibre was rotated to find the position, where the spectrum shift is the same regardless of whether the displacement is on the + x-axis or on the –x-axis (Figs. 6a, 7a). Then, by rotating the MMCF ± 90º from the reference position (see Fig. 5b), the written core is placed in such a way that it lies in the bend plane, so the fibre index profiles for left and right bends are no longer identical. Thus, according to the Eq. (4), positive or negative bending on the fibre will cause the largest spectral shifts to shorter or larger wavelengths (see Figs. 6b, 7b–d) enabling to detect the magnitude and direction of the bending.

After locating the desired position of the fibre, we experimentally evaluated its performance. The MMFC was displaced by means of the stepper motor from the straight position in the direction of the + *x*-axis in steps of 0.5 mm until reaching 5 mm. Next, it was returned to the straight position in the same steps. The latter operation was repeated on the − *x*-axis direction (Fig. 7b,c). In these graphics we have converted the displacement distance to degrees and it can be seen how when the bending causes expansion in the written core (as it is illustrated in Fig. 4b the spectrum red-shift and on the contrary, when the bending causes contraction, the spectrum blue-shift. In Fig. 7d we have plotted the wavelength shift versus the bend angle. From this calibration, we found that the sensitivity of our sensor for positive and negative bending is − 1.4 nm/° and 1 nm/°, respectively. The error bars represent the range in which are found the return from the maximum bent angle to straight position. From the width of the error bars it can be seen that our proposed vector-bend sensor does not exhibit hysteresis.

The ability to distinguish the bend direction can also be understood by analogy. Consider a symmetric MCF under bending, in which one side is under compression and the other tension. If the MCF is initially straight, due to geometric symmetry, the spectral peak wavelength shift is identical whether the MCF is bent to the left or right, so orientation is unresolvable. However, if the MCF began from an initially curved position, it would be able to resolve whether the bend has become tighter or weaker due to different peak shift direction. By modifying the index of an outer core of the MCF, we effectively 'bias' our MCF sensor into this regime of operation: the permanent laser-induced index increase here simulates the compression experienced on the inner bend and has a similar effect on the supermode profiles. In this way, the asymmetric MMCF can resolve bend direction from a straight initial position.

**Figure 8:**
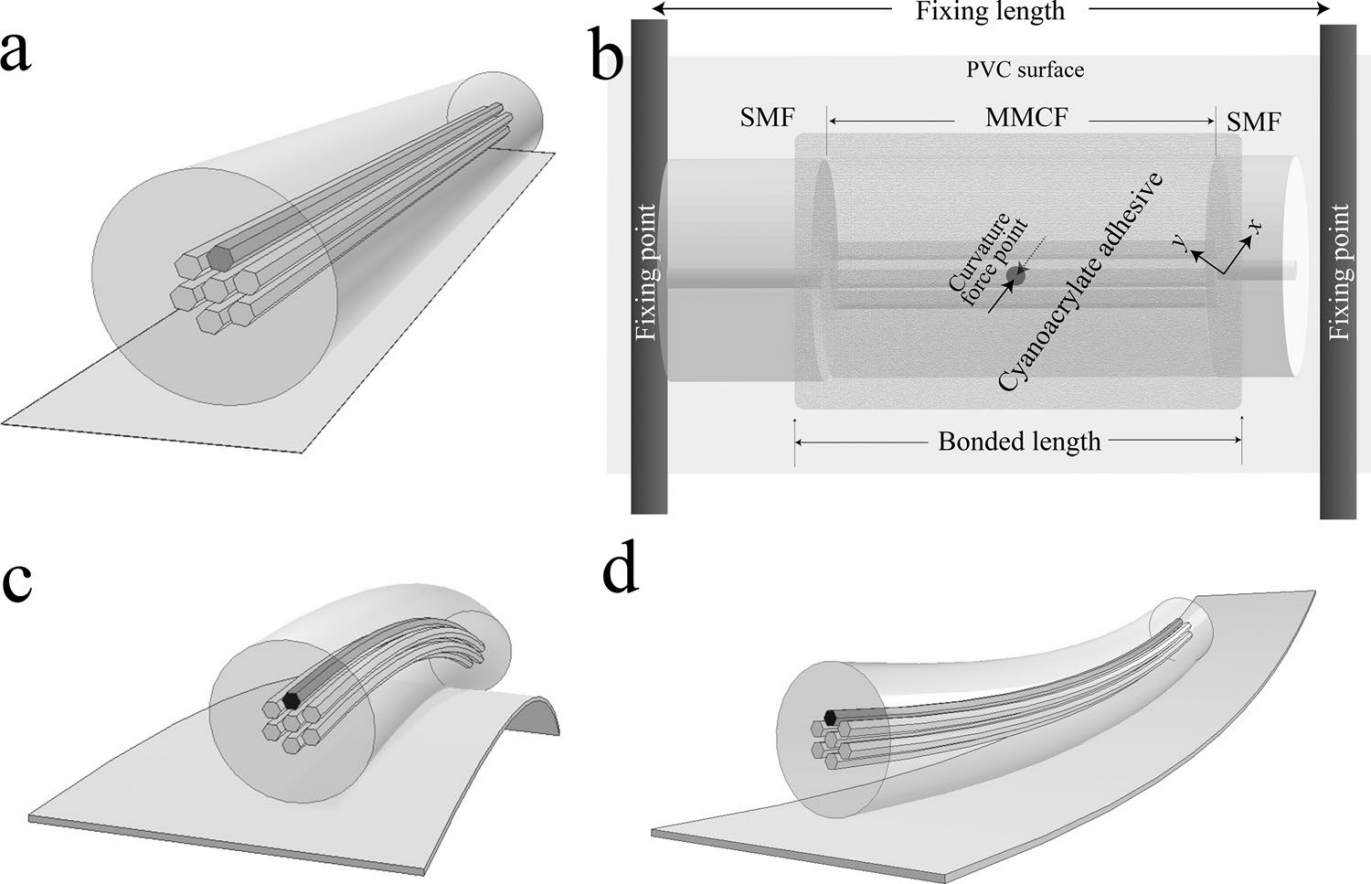
(**a**) Illustration of the distribution of the cores to measure curvature. (**b**) Setup used to measure the curvature experimentally. (**c**,**d**) Illustration of the expansion and compression, respectively, of the written core depending on the direction of the curvature.

Our proposed sensor was also evaluated as a directional curvature sensor. To carry out the experimental test, first we placed the core distribution of a length of ~ 4 cm of MMCF symmetrically with respect to the *y*-axis using the same technique as described above. Afterwards, a sheet of PVC was placed underneath the MMCF and bonded together (Fig. 8a). In order to transmit the curvature from the PVC sheet to the MMCF segment, the MMCF was covered along the whole length with a cyanoacrylate adhesive (Fig. 8b) and left to cure for 24 h.

Once the device was bonded to the PVC, it was vertically placed and fixed by two points with a fixing length of 10 cm (see Fig. 8b). Next, by means of the motorised flexure stage, it was subjected to displacements of 5 mm in steps of 0.5 mm in on the ± *x*-axis direction, applying a force at the curvature force point as described in the Fig. 8b. It causes tension on the written core when the displacement is on the + *x* direction and compression when the – *x*-direction. Taken into account the fixing length we have converted displacements to curvature (m^−1^) (Fig. 8).

**Figure 9:**
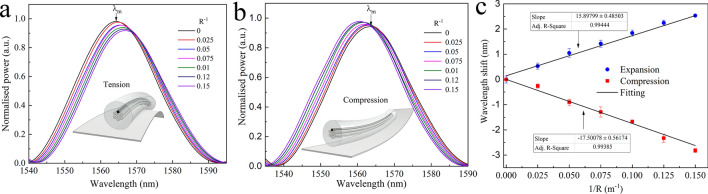
(**a**,**b**) Experimental spectra obtained from the MMCF device when it was subjected to different direction of curvature. (**c**) Plot of peak wavelength shift Δλ_m_ against curvature for both directions.

From the data (Fig. 9), it can be seen that the wavelength peak is red-shifted when the laser modified core undergoes tension (positive curvature) and blue-shifted when the core compresses (negative curvature). The error bars represent the shift range in which are found when the curvature return from its maximum to straight position. From the calibration we found that our device has a sensitivity of 15.9 and − 17.5 nm/m^−1^ to positive and negative curvature, respectively. Once again, it can be seen that the device does not exhibit hysteresis.

Our device also was assessed in a static test of an aeronautical component made of carbon fibre. This component was developed by Aernnova, a company specialising in the design and manufacturing of aeronautical structures and components. The test consisted of a Static Test at room temperature carried out at the Aeronautical Technology Centre (CTA) placed in Miñano, Spain. The test consisted of applying a load to the specimen using hydraulic actuators controlled by a workstation (MTS Systems Corporation). The applied load causes bending deformation of the specimen. In parallel, the deformation was measured by strain gauges (HBM, Germany) placed at different points.

**Figure 10:**
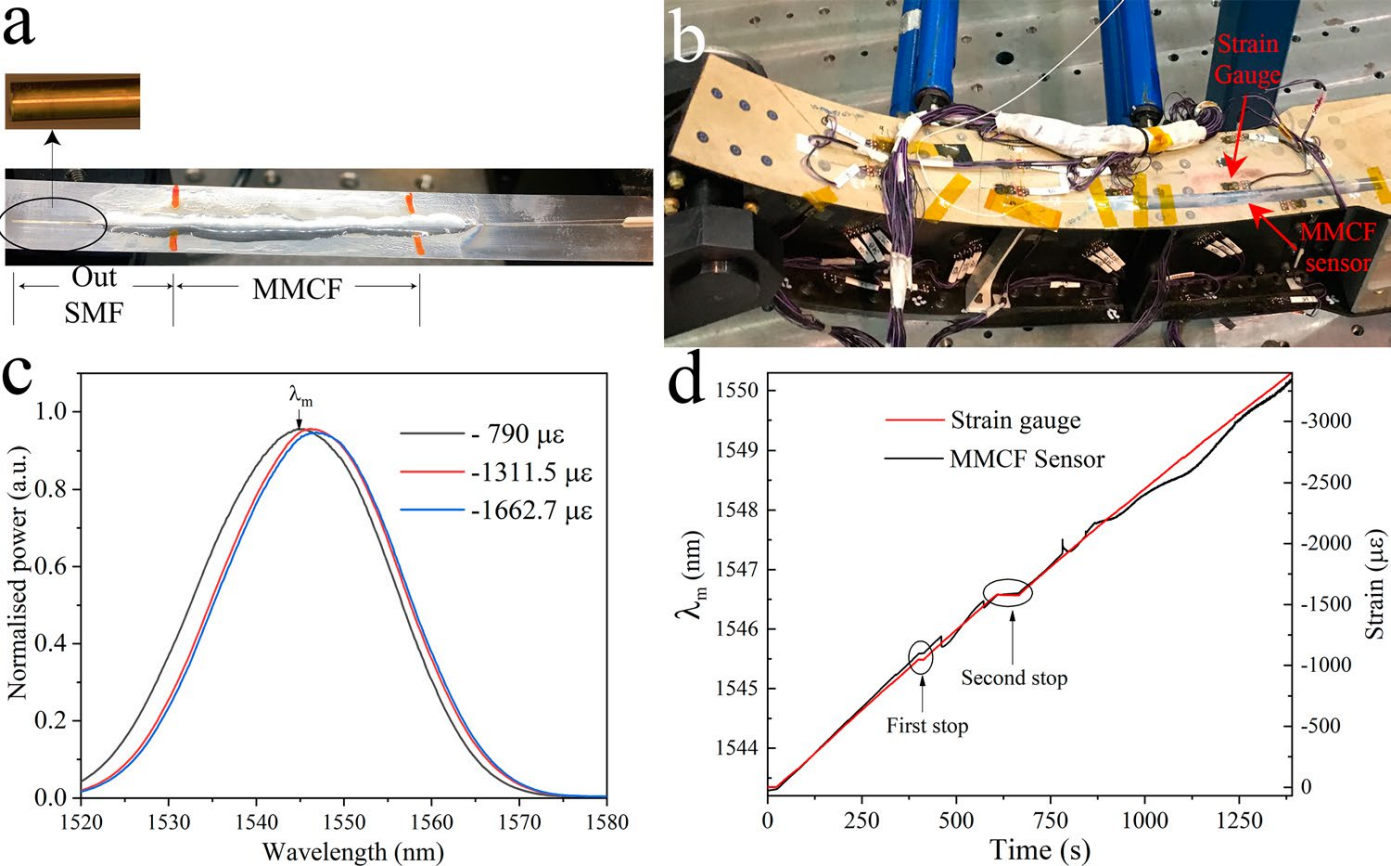
(**a**) Image of the structure formed by the MMCF device and the Thickness Gauge Foil. Inset shows the gold-coated out-SMF. (**b**) Image of our device and the strain gauge placed in the aeronautical structure. (**c**) Experimental spectrum showing peak wavelength shift as the strain increases. (**d**) Comparison of our device with the commercial strain gauge.

Our device was bonded to a thickness gauge foil (TGF) of 0.05 mm (Vogel, Germany) with the cores in the same way as shown in Fig. 8a. The tip of the out-SMF was coated with a 100 nm thick gold layer by means of a sputter coater (see Fig. 10a). The gold coating acts as a mirror and protects from the environmental dirt.

In order to compare our proposed device with commercial strain sensors, the structure formed with the TGF and our device was glued with cyanoacrylate to the specimen in the vicinity of the strain gauges installed by the CTA technicians (see Fig. 10b). The setup for carrying out such test is as shown in Fig. 3b. A superluminescent diode (SLD) was used as a broadband light source centred at 1,550 nm. The spectrum was collected in real-time by means of a miniature interrogation monitor (Ibsen Photonics, Denmark) (Fig. 10c). To measure the deformation of the specimen at the point where we placed our device, the local peak of the spectrum (λ_m_) was tracked.

The real-time evolution of the test, while the specimen was subjected from 0 to 3,300 micro-strain (με), can be seen in Fig. 10d. The force was applied to the specimen continuously with two stops made at 1,000 and 1,500 με (labelled in the Fig. 10d as “first stop” and “second stop”). From the aforementioned figure it can be seen that our device can detect the evolution of the deformation of the specimen according to the applied force just like the commercial strain gauge with which it has been compared. From around 2000 με, the spectral peak shift occurs in a more abrupt fashion. From the recorded videos of the test, we have identified that these abrupt shifts are caused by the delamination of the layers of the specimen at this area. It is important to note that the area chosen for placing our sensor was predicted to fail by the theoretical simulations when the load reaches ~5,000 με. Note that our device covers a wider area compared to the area covered by the strain gauge.

## Discussion

We have proposed and experimentally demonstrated a vector bending and distinguishing curvature orientation sensor based on coupling between the 7 cores of a multi-core fibre. By breaking the refractive index symmetry of the cores, bend direction can be distinguished. The measured sensitivities of − 17.5 nm/m^−1^ and − 1.4 nm/° as a curvature and a vector bending sensor, respectively, are higher than any other reported so far. The sensor has the advantages of high repeatability, low-cost, straightforward and reproducible fabrication, and does not have hysteresis. When our sensor was evaluated in a static test of an aeronautical component, made of carbon, its behaviour successfully matched that of a commercial sensor.

## Methods

Our femtosecond laser system Pharos (Light Conversion Ltd., Lithuania) operates at 1030 nm wavelength, with a pulse duration of 200 fs and a repetition rate of 200 kHz. For enhancing writing resolution, laser writing was carried out using the second harmonic at 515 nm. The laser beam was focused by a 0.4 NA objective into the bulk of the MCF, which was fixed onto a computer controlled translation stage. An iris with 65% transmission was placed before the objective to improve the beam homogeneity. During the writing, the laser writing power was set to 6 mW after the objective, which corresponds to a single laser pulse energy of 30 nJ. The modification was written by the multiscan laser scanning method^35^ where the laser scanlines were inscribed consecutively one next to the other, separated laterally by 200 nm, and the pulse density was 3 × 10^5^ pulses/mm along each scanline.
