# Author Correction: High-performance vector bending and orientation distinguishing curvature sensor based on asymmetric coupled multi-core fibre

**DOI:** 10.1038/s41598-020-80648-9

**Published:** 2021-01-11

**Authors:** Oskar Arrizabalaga, Qi Sun, Martynas Beresna, Timothy Lee, Joseba Zubia, Javier Velasco Pascual, Idurre Sáez de Ocáriz, Axel Schülzgen, Jose Enrique Antonio‑Lopez, Rodrigo Amezcua‑Correa, Joel Villatoro, Gilberto Brambilla

**Affiliations:** 1 Department of Communications Engineering, University of the Basque Country (UPV/EHU), Ingeniero Torres Quevedo s/n, 48013 Bilbao, Spain; 2 Optoelectronics Research Centre, University of Southampton, Southampton, SO17 1BJ UK; 3 Fundación Centro de Tecnologías Aeronáuticas (CTA), Miñano, Spain; 4 The College of Optics and Photonics, CREOL, University of Central Florida, Orlando, USA; 5 IKERBASQUE-Basque Foundation for Science, 48011 Bilbao, Spain

Correction to: *Scientific Reports* 10.1038/s41598-020-70999-8, published online 20 August 2020

This Article contains an error in the Reference list. The authors omitted the below paper, which is shown as Reference 1. This should be cited in the Results section as below:

“In the case of the bent MMCF, the fibre elongates/compresses at the circular bend and the local index changes because of stress-optic effect upon bending. The index transformation is given as follows^22,34^:”

should read:

“In the case of the bent MMCF, the fibre elongates/compresses at the circular bend and the local index changes because of stress-optic effect upon bending. The index transformation is given as follows^22,1^:”

Additionally, Equation 5 contains errors.

$$ n^{\prime}(x,y) = n(x,y)\left[ {1 - \frac{{n(x,y)^{2} \,x\,}}{2R}\left( {P_{12} - \nu \left( {P_{11} + P_{12} } \right)} \right)} \right]\,\exp \left( \frac{x}{R} \right) $$

should read:

$$ n^{\prime}\left( {x,y} \right) = n\left( {x,y} \right)\left\lfloor {1 - \frac{{n\left( {x,y} \right)^{2} }}{2R}\left( {P_{12} - \left( {P_{11} + P_{12} } \right)} \right)1 + \frac{x}{R}} \right\rfloor $$

Finally, this Article contains an error in the legend of Figure 2:

“(**b**) Experimental setup. *SCLS* super continium light source.”

should read:

“(**b**) Experimental setup. *SCLS* denotes supercontinuum light source.”
